# Changes of overweight and obesity in the adult Swiss population according to educational level, from 1992 to 2007

**DOI:** 10.1186/1471-2458-10-87

**Published:** 2010-02-22

**Authors:** Pedro Marques-Vidal, Pascal Bovet, Fred Paccaud, Arnaud Chiolero

**Affiliations:** 1 Institute of Social and Preventive Medicine (IUMSP), University Hospital Center (CHUV) and University of Lausanne, Lausanne, Switzerland; 2 Cardiomet, University Hospital Center (CHUV), Lausanne, Switzerland

## Abstract

**Background:**

In many high income developed countries, obesity is inversely associated with educational level. In some countries, a widening gap of obesity between educational groups has been reported. The aim of this study was to assess trends in body mass index (BMI) and in prevalence of overweight and obesity and their association with educational level in the adult Swiss population.

**Methods:**

Four cross-sectional National health interview surveys conducted in 1992/93 (n = 14,521), 1997 (n = 12,474), 2002 (n = 18,908) and 2007 (n = 17,879) using representative samples of the Swiss population (age range 18-102 years). BMI was derived from self-reported data. Overweight was defined as BMI ≥ 25 and <30 kg/m^2^, and obesity as BMI ≥ 30 kg/m^2^.

**Results:**

Mean (± standard deviation) BMI increased from 24.7 ± 3.6 in 1992/3 to 25.4 ± 3.6 kg/m^2 ^in 2007 in men and 22.8 ± 3.8 to 23.7 ± 4.3 kg/m^2 ^in women. Between 1992/3 and 2007, the prevalence of overweight + obesity increased from 40.4% to 49.5% in men and from 22.3% to 31.3% in women, while the prevalence of obesity increased from 6.3% to 9.4% in men and from 4.9% to 8.5% in women. The rate of increase in the prevalence of obesity was greater between 1992/3 and 2002 (men: +0.26%/year; women: +0.31%/year) than between 2002 and 2007 (men: +0.10%/year; women: +0.10%/year). A sizable fraction (~25%) of the increasing mean BMI was due to increasing age of the participants over time. The increase was larger in low than high education strata of the population. BMI was strongly associated with low educational level among women and this gradient remained fairly constant over time. A weaker similar gradient by educational level was apparent in men, but it tended to increase over time.

**Conclusion:**

In Switzerland, overweight and obesity increased between 1992 and 2007 and was associated with low education status in both men and women. A trend towards a stabilization of mean BMI levels was noted in most age categories since 2002. The increase in the prevalence of obesity was larger in low education strata of the population. These findings suggest that obesity preventive measures should be targeted according to educational level in Switzerland.

## Background

The association between educational level and obesity varies between populations [1]. Several studies in the USA [2] and in European countries have shown a widening gap in obesity levels between socio-economic groups [3-5].

On the other hand, because obesity is increasingly frequent in every socio-economic stratum, it may be possible that the difference in the obesity prevalence between socio-economic strata has decreased, if not vanished. In the USA, one study suggested that differences in the prevalence of obesity by socio-economic status had decreased [6,7]. One study conducted in Geneva, Switzerland, suggested converging prevalences of obesity across educational groups [8]. However, this study was based on data collected in one town and may not reflect national trends.

Using national representative data from all available Swiss Health Surveys (SHS), we assessed the trends in mean BMI, overweight and obesity in Switzerland between 1992 and 2007 and evaluated whether the association between obesity and SES has changed during this period.

## Methods

### Swiss Health Survey

Data for the four Swiss Health Surveys (SHS) were obtained from the Swiss federal bureau of statistics http://www.bfs.admin.ch. The SHS is a cross-sectional, nationwide, population-based telephone survey conducted every 5 years since 1992 by the Federal Statistical Office of Switzerland under a mandate of the Federal Government [9]. The SHS aims to track public health trends in a representative sample of the resident population of Switzerland aged 15 and over. To date the survey has been carried out four times, in 1992/93, 1997, 2002 and 2007.

The study population was chosen by stratified random sampling of a database of all private Swiss households with fixed line telephones. It is currently estimated that over 90% of the Swiss households have fixed telephones. The first sampling stratum consisted of the seven main regions: West "Leman", West-Central "Mittelland", Northwest, Zurich, North-Eastern, Central, and South. The second stratum consisted of the cantons, and the number of households drawn was proportional to the population of the canton. In some cantons, oversampling of the households was made to obtain accurate cantonal estimates, and extra strata were used for the cantons of Zurich and Bern. Overall, 29 strata were used. Within these 29 strata, households were randomly drawn and, within the household, one member was randomly selected within all members aged 15 years and over.

A letter inviting one specific household member to participate in the survey was sent to each sampled subject, who was contacted thereafter by phone and interviewed using computer-assisted telephone interview software to manage dialling and data collection. Face-to-face interviews were organised for subjects older than 75 years. In the case of long-term absence of a sampled subject, a proxy interviewee was requested to provide answers on behalf of the pre-defined sampled person. The interviews were carried out in German, French or Italian, as appropriate. People who did not speak any of these three languages were excluded from the survey. Other criteria for exclusion were: asylum seeker status, very poor health status, and living in a nursing home [10]. Four sampling waves were performed (winter, spring, summer, and autumn). Participation rate was 71% in 1992/93, 85% in 1997, 64% in 2002, and 66% in 2007. More details are available at http://www.bfs.admin.ch/bfs/portal/fr/index/infothek/erhebungen__quellen/blank/blank/ess/01.html.

### Data collected

Current body weight and height were self-reported. Overweight and obesity were defined for a BMI ≥ 25 and <30, and ≥ 30 kg/m^2^, respectively.

Three age categories were considered: 18-44, 45-64 and 65+ years. Education was categorized as follows: 1) no education completed; 2) primary school; 3) lower secondary level; 4) upper secondary level, and 5) tertiary level, which included university and other forms of education after the secondary level. As educational level 1 was very low (1% in men and 1.6% in women), we defined educational level as "low" (categories 1 and 2), "middle" (categories 3 and 4), and "high" (category 5) groups.

### Statistical analysis

Statistical analysis was conducted using SAS Enterprise Guide version 4.1 (SAS Institute Inc., Cary, NC, USA). Prevalence of overweight and/or obesity and mean BMI (± standard deviation) were calculated by age and educational categories for each survey. A first analysis was conducted using the original data from the surveys, and a second analysis was conducted after weighting each subject according to the formula

Where N_*h *_is the average number of telephone numbers in stratum *h *(h = 1 to 29), H_*i *_is the household size, i.e. the number of subjects aged 15 and over living in household *i*, and n_*h *_is the number of telephone numbers in the sample S_*h *_corresponding to stratum *h*. Weights tool also into account the percentage of nonresponders by raking ratio estimation [11]. Further, as height and weight were self-reported, a possible underestimation of obesity prevalence could occur. Hence, the prevalence of overweight and obesity were also assessed correcting for the bias reported in a previous study conducted in Switzerland [12], i.e., adding 0.8 kg/m^2 ^and 1.1 kg/m^2 ^to the BMI values of men and women, respectively. A second correction was performed using gender- and age-specific values, according to [13], i.e. +0.18 and +0.58 kg/m^2 ^for men and women aged <60 years, respectively, and +0.64 and +1.05 kg/m^2 ^for men and women aged ≥60 years, respectively. Trends in BMI over time (slope ± standard error) were assessed using linear regression. Comparisons between surveys were performed using Chi-square or analysis of variance. Adjustment for age was performed using a general linear model. Comparisons between groups were adjusted for survey using Cochran-Mantel-Haenszel test. Factors related to the prevalence of obesity were assessed separately for each gender by multivariate logistic regression adjusting for survey, age group, education, nationality (Swiss vs. non-Swiss) and smoking status (never, former, current). Statistical significance was assessed for p < 0.05.

## Results and discussion

### Results

Characteristics of the participants according to survey year and sex are summarized in Table 1. Over successive surveys, the proportion of participants with low educational level decreased and the proportion of participants with high educational level increased. Mean age, BMI and the prevalence of overweight and obesity increased over surveys in both sexes and for all age groups (table 2). Similar findings were observed after weighting or correcting for self-report bias (table 3). The prevalence of overweight was higher in men than in women, whereas the prevalence of obesity was slightly higher in men than in women.

**Table 1:** Characteristics of the participants and prevalence of overweight and obesity in the successive surveys.

	1992/3	1997	2002	2007	Test
Sample size	14,521	12,474	18,908	17,879	3.16 ^NS^
Women (%)	7946 (54.7)	6937 (55.6)	10,345 (54.7)	9862 (55.2)	
Age (years)					
Men	44.3 ± 16.7	45.2 ± 17.0	48.6 ± 16.9	49.6 ± 17.2	160.2 ***
Women	46.1 ± 17.5	47.8 ± 18.1	50.4 ± 17.3	51.3 ± 17.9	160.5 ***
Educational level (%)					
Low	3081 (21.2)	2747 (22.0)	3635 (19.2)	2471 (13.8)	
Middle	8300 (57.2)	7577 (60.7)	12,082 (63.9)	10,523 (58.9)	1005 ***
High	3140 (21.6)	2150 (17.2)	3191 (16.9)	4885 (27.3)	
Men					
Mean BMI (kg/m^2^)	24.7 ± 3.6	24.9 ± 3.4	25.3 ± 3.7	25.4 ± 3.6	65.06 ***
Overweight (%)	5841 (34.1)	1980 (35.8)	3351 (39.1)	3217 (40.1)	184.9 ***
Obesity (%)	411 (6.3)	374 (6.8)	765 (8.9)	752 (9.4)	
Women					
Mean BMI (kg/m^2^)	22.8 ± 3.8	23.4 ± 4.3	23.7 ± 4.2	23.7 ± 4.3	90.95 ***
Overweight (%)	1386 (17.4)	1470 (21.2)	2398 (23.2)	2248 (22.8)	231.6 ***
Obesity (%)	392 (4.9)	500 (7.2)	825 (8.0)	836 (8.5)	
All subjects					
Mean BMI (kg/m^2^)	23.6 ± 3.8	24.0 ± 4.0	24.4 ± 4.1	24.5 ± 4.1	
Overweight (%)	3627 (25.0)	3450 (27.7)	5749 (30.4)	5465 (30.6)	386.4 ***
Obesity (%)	803 (5.5)	874 (7.0)	1590 (8.4)	1588 (8.9)	

BMI: body mass index; overweight is defined as a BMI ≥ 25 and <30 kg/m^2^. Obesity is defined as a BMI ≥ 30 kg/m^2^. Results are expressed as number of subjects and (percentage) or as mean ± standard deviation. Statistical analysis by chi-square or analysis of variance: ^NS^, not significant; ***, p < 0.001. For BMI, further adjustment on age led to similar findings: F test = 28.8 (p < 0.001) for men and F test = 44.1 (p < 0.001) for women.

**Table 2:** Prevalence of overweight and obesity, by survey year, sex and age group.

Gender/age	1992	1997	2002	2007
**Men**				
Overweight (%)				
[18-44]	976 (26.6)	807 (27.0)	1283 (32.4)	1157 (33.3)
[45-64]	857 (44.5)	767 (47.4)	1251 (44.0)	1258 (44.8)
[65+	408 (41.7)	406 (44.0)	817 (46.7)	802 (46.0)
Obesity (%)				
[18-44]	150 (4.1)	132 (4.4)	219 (5.5)	220 (6.4)
[45-64]	180 (9.3)	155 (9.6)	344 (12.1)	331 (11.8)
[65+	81 (8.3)	87 (9.4)	202 (11.5)	201 (11.5)
**Women**				
Overweight (%)				
[18-44]	396 (9.7)	449 (13.2)	640 (14.8)	586 (14.7)
[45-64]	541 (22.5)	539 (26.9)	906 (25.8)	782 (24.0)
[65+	449 (30.2)	482 (31.4)	852 (34.0)	880 (33.5)
Obesity (%)				
[18-44]	101 (2.5)	128 (3.8)	212 (4.9)	211 (5.3)
[45-64]	167 (7.0)	191 (9.6)	323 (9.2)	319 (9.8)
[65+	124 (8.4)	181 (11.8)	290 (11.6)	306 (11.7)

Non-weighted data.

Overweight is defined as a BMI ≥ 25 and <30 kg/m^2^. Obesity is defined as a BMI ≥ 30 kg/m^2^. Results are expressed as number of subjects and (percentage). Statistical analysis adjusting for survey by Cochran-Mantel-Haenszel test: for men, test = 893.6, p < 0.001; for women, test = 1665.5, p < 0.001.

**Table 3:** Prevalence of overweight and obesity by survey year and sex.

Gender	1992	1997	2002	2007
**Men**				
Overweight (%)				
Uncorrected, weighted	34.2	36.8	39.3	39.5
Corrected ^a^, unweighted	43.4	44.6	47.5	47.9
Corrected ^a^, weighted	44.2	45.4	47.5	47.1
Corrected ^b^, unweighted	36.8	38.7	42.1	43.0
Corrected ^b^, weighted	37.2	39.9	42.1	42.2
Obesity (%)				
Uncorrected, weighted	6.3	7.0	8.3	9.0
Corrected ^a^, unweighted	8.8	9.8	12.0	12.4
Corrected ^a^, weighted	8.8	10.1	11.3	11.9
Corrected ^b^, unweighted	7.2	7.9	10.1	10.6
Corrected ^b^, weighted	7.3	8.1	9.2	10.0
**Women**				
Overweight (%)				
Uncorrected, weighted	17.8	21.8	22.7	21.7
Corrected ^a^, unweighted	24.1	26.9	28.9	28.3
Corrected ^a^, weighted	24.1	28.0	28.4	27.1
Corrected ^b^, unweighted	22.1	25.0	27.5	26.6
Corrected ^b^, weighted	22.1	25.9	26.6	25.3
Obesity (%)				
Uncorrected, weighted	4.9	7.2	7.8	8.1
Corrected ^a^, unweighted	6.6	10.2	11.1	11.6
Corrected ^a^, weighted	6.7	10.2	10.8	11.0
Corrected ^b^, unweighted	5.9	9.1	9.8	10.6
Corrected ^b^, weighted	5.8	9.1	9.5	9.9

Date were weighted or corrected for self-report bias.

Overweight is defined as a BMI ≥ 25 and <30 kg/m^2^. Obesity is defined as a BMI ≥ 30 kg/m^2^. Results are expressed as percentage, obtained using original sample data weighted to reflect the Swiss population (uncorrected, weighted), BMI corrected for reporting bias (corrected, unweighted) and BMI corrected for reporting bias and weighted to reflect the Swiss population (corrected, weighted). ^a ^gender-specific correction; ^b^, gender and age-specific correction.

The rate of increase in the prevalence of obesity was larger between 1992 and 2002 (men: +0.26%/year; women: +0.31%/year) than between 2002 and 2007 (men: +0.10%/year; women: +0.10%/year) and similar findings were obtained after correcting for self-report bias and weighting (not shown). After adjusting for age, the increase over time in mean BMI was reduced: from 24.8 ± 0.1 (adjusted mean ± standard error) in 1992/3 to 25.3 ± 0.1 kg/m^2 ^in 2007 in men, and from 23.0 ± 0.1 to 23.6 ± 0.1 kg/m^2 ^in women. Overall, the increase in mean age during the study period explained 28% of the increase in mean BMI values in men and 33% in women. Mean BMI increased over time more in the younger than older participants: in men, the linear secular difference (± standard error) of mean BMI per calendar year was 0.052 ± 0.005 and 0.023 ± 0.005 kg/m^2^/year in subjects aged <45 and ≥45 years, respectively (p < 0.001). In women, the corresponding values were 0.066 ± 0.005 and 0.026 ± 0.006 kg/m^2^/year (p < 0.001).

**Figure 1:**
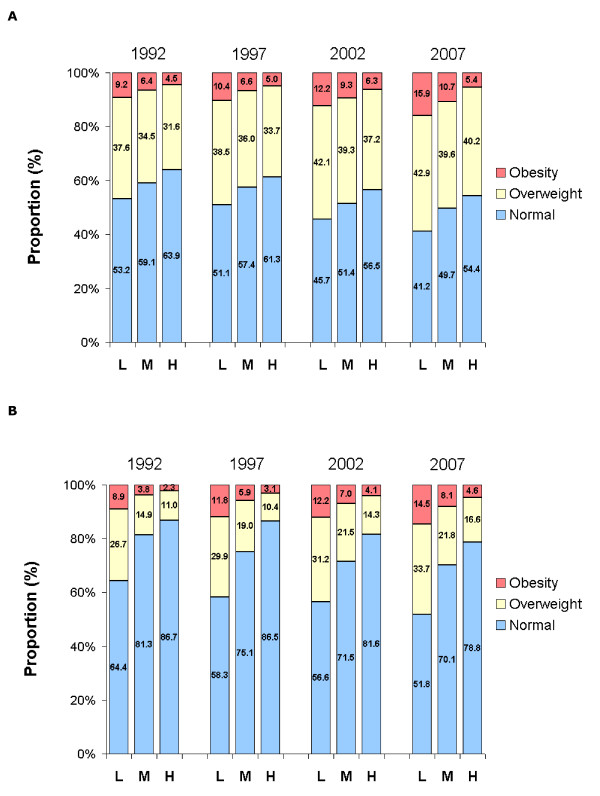
**Prevalence of body mass index categories according to sex, survey year and educational level**. Panel A, men, panel B, women. Educational level: L, low; M, middle; H, high.

**Figure 2:**
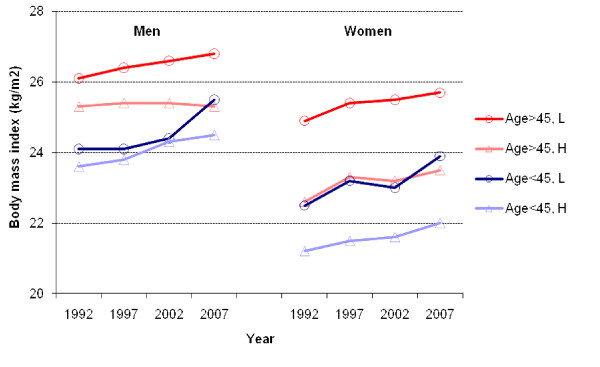
**Mean body mass index according to sex, survey year, age and low or high educational level**. (Mean BMI for the middle educational level was intermediary between upper and lower educational levels and is not displayed for clarity).

In each survey, the prevalence of overweight and obesity was inversely associated with educational level, and this association was more marked in women than in men (Figure 1). The yearly increase in the prevalence of obesity was +0.45%, +0.29% and +0.06% among men with low, middle or high educational level, respectively, the corresponding increases for women being +0.37%, +0.29% and +0.15% (Table 4). The educational gradient in BMI was fairly constant over time in women, while it tended to increase over the last years in men (Figure 2). Mean BMI for middle educational level was intermediary between upper and lower educational levels and is not displayed for clarity of the figure. Similar results were obtained after correction for self-reported bias or weighting (not shown).

**Table 4:** Prevalence of overweight and obesity, by survey year, sex and educational group.

Gender/educational level	1992	1997	2002	2007
**Men**				
Overweight (%)				
Low	368 (37.6)	332 (38.5)	470 (42.1)	316 (42.9)
Middle	1252 (34.5)	1141 (36.0)	2074 (39.4)	1794 (39.6)
High	621 (31.6)	507 (33.6)	807 (37.1)	1107 (40.3)
Obesity (%)				
Low	90 (9.2)	90 (10.4)	136 (12.2)	117 (15.9)
Middle	233 (6.4)	208 (6.6)	493 (9.3)	486 (10.7)
High	88 (4.5)	76 (5.1)	136 (6.3)	149 (5.4)
**Women**				
Overweight (%)				
Low	561 (26.7)	564 (29.9)	786 (31.2)	585 (33.7)
Middle	695 (14.9)	839 (19.0)	1466 (21.5)	1309 (21.9)
High	130 (11.1)	67 (10.4)	146 (14.3)	354 (16.6)
Obesity (%)				
Low	188 (8.9)	222 (11.8)	307 (12.2)	251 (14.5)
Middle	177 (3.8)	258 (5.9)	476 (7.0)	486 (8.1)
High	27 (2.3)	20 (3.1)	42 (4.1)	99 (4.6)

Non-weighted data.

Overweight is defined as a BMI ≥ 25 and <30 kg/m^2^. Obesity is defined as a BMI ≥ 30 kg/m^2^. Results are expressed as number of subjects and (percentage). Statistical analysis adjusting for survey by Cochran-Mantel-Haenszel test: for men, test = 7.32, p < 0.01; for women, test = 1064, p < 0.001.

Finally, multivariate analysis showed higher education to be significantly related with a lower odds of presenting obesity in both genders (table 5).

**Table 5:** multivariate analysis of determinants of obesity in the Swiss population

	Men	Women
Study year		
1992-3	1 (ref.)	1 (ref.)
1997	1.07 [0.92-1.24]	1.44 [1.26-1.66]
2002	1.38 [1.22-1.56]	1.57 [1.39-1.78]
2007	1.51 [1.33-1.71]	1.79 [1.57-2.03]
Age group		
(18-44)	1 (ref.)	1 (ref.)
(45-64)	2.11 [1.91-2.34]	2.01 [1.81-2.23]
(65+	1.83 [1.62-2.07]	2.19 [1.95-2.45]
Education		
Lower	1 (ref.)	1 (ref.)
Middle	0.72 [0.64-0.81]	0.59 [0.53-0.64]
High	0.43 [0.37-0.49]	0.35 [0.30-0.41]
P-value for trend	<0.001	<0.001
Nationality		
Swiss	1 (ref.)	1 (ref.)
Non-Swiss	1.01 [0.89-1.15]	1.16 [1.02-1.32]
Smoker		
Never	1 (ref.)	1 (ref.)
Current	1.11 [1.00-1.23]	0.79 [0.70-0.88]
Former	1.45 [1.30-1.61]	1.26 [1.14-1.40]

Obesity is defined as a BMI ≥ 30 kg/m^2^. Statistical analysis by logistic regression. Results are expressed as Odds-ratio and [95% confidence interval].

### Discussion

In Switzerland, mean BMI levels and the prevalence of obesity increased between 1992 and 2007. A sizable fraction (~25%) of the increasing mean BMI was due to increasing age of the participants over time. However, a trend towards a stabilization of mean BMI levels was noted in most age and educational categories since 2002, except for young persons of low educational level. The ongoing increase of mean BMI among the younger age groups (particularly among persons of low educational level) is a major concern and might actually reflect the recent increase in child and adolescent obesity levels in Switzerland [14]. In view of longer life span, upward trends in BMI in younger persons have a large potential for long-term detrimental health impact. However, the prevalence of obesity remained relatively low in Switzerland compared to other Europeans countries [15], and comparison of the results from this study with other data according to survey year, age group and gender led to similar findings (additional files 1, 2 and 3).

Educational level was strongly and inversely associated with the prevalence of a BMI ≥ 25 kg/m^2 ^and obesity. These findings are in agreement with the literature (for a review, see [16]), and possible explanations include differences in dietary habits and physical activity levels. Indeed, several studies have shown that subjects in higher socioeconomic groups tend to have a healthier dietary intake, with an increased consumption of fruits, vegetables and low fat foods, whereas subjects with lower economic capacity favour cheaper foods with a higher fat and caloric content [17-20]. Regarding physical activity, subjects in lower status occupations are less likely to report participation in vigorous leisure-time physical activity sufficient for cardiorespiratory fitness [21], and a decrease in physical activity demand in the workplace [22] associated with a decreased access to sport facilities due to cost [23,24], shift work [25] or environmental constraints [26-28] might also intervene.

We also observed that the association between educational level and obesity remained strong between 1992 and 2007 in women and may have increased in younger men. The widening of the BMI difference between the high and low educational groups in men is a matter of concern, and this has also been reported in France [29,30] but not in some other countries [2,4,31]. Our findings contrast with a previous study in Geneva [8]: in this study the upward secular trends in the prevalence of obesity tended to be similar within each education stratum in women (consistent with our findings), while the BMI difference by educational level tended to decrease over time. In Switzerland, we previously showed that children whose parents had low educational level were more frequently obese [14]. Overall, our data indicate that trends in BMI ≥ 25 kg/m^2 ^and trends in obesity might be diverging according to socioeconomic status in Switzerland, and that preventive measures against obesity should be targeted accordingly.

A slowing of the increase in the prevalence of obesity was found between 2002 and 2007. Those findings are partly in agreement with a previous study that showed a levelling off of obesity in women [32]. Possible explanations include the increase in the prevalence of subjects doing exercise [33] or changes in diet [34,35], although the former are of too low magnitude to significantly impact weight. Still, the perceived importance of diet and physical activity as priorities for the prevention in the general population increased consistently between 1996 and 2006 [36]. Another likely explanation is that non-responders tend to present with a higher BMI, but this was found only among women [37] and thus cannot explain the trends in men.

This study has some limitations. Height and weight were self-reported, leading to an underestimation of obesity prevalence. With a correction of self-report bias, much larger prevalence of overweight and obesity were found. However, trends were not changed. Based on corrected and weighted data, it was estimated that, in Switzerland, circa 47.1% of men and 27.1% of women are overweight, the corresponding figures for obesity being 11.9% and 11.0%, when the biggest correction was applied, while the use of gender- and age-specific corrections led to lower prevalence rates. Those values are close to those obtained in a population based study in Lausanne (CoLaus study) using measured height and weight, although the prevalence of obesity was higher in the CoLaus study [38]. Possible explanations for the difference regarding obesity prevalence are the fact that the CoLaus study only included Caucasian subjects living in the city of Lausanne and aged between 35 and 75 years; indeed, restricting the analysis to subjects of this age group considerably reduced the difference (prevalence of overweight+obesity in 2007: 65.2% and 42.5% in men and women, respectively, vs. 72.4% and 42.6% in the CoLaus study). Finally, it should be noted that, according to a recent review comparing self-reported and measured data [39], it is possible that the magnitude of the underestimation of BMI values using self-reported data decreases with time, albeit the available data come from different studies conducted in different countries. Hence, the underestimation of the prevalence of overweight/obesity for the 1992-3 survey might be higher than for the 2007 survey, suggesting that the increase in the prevalence of overweight/obesity observed might actually be lower than reported. Still, to our knowledge, there is no consistent data regarding the evolution of BMI underestimation with time and further studies are needed to better assess this point.

The participation rate decreased slightly between surveys. Although the decreasing participation rate might be an issue, still it was high compared to other studies [40,41], and the magnitude of the non-participation bias may not be proportional to the percentage of non-participants [42]. The results were similar with and without weighting, indicating the absence of a significant bias that might have distorted the results. The major advantages of this study is that it is based in nationally representative samples, and that the data has been collected using the same methodology throughout time, thus enabling assessing trends with confidence.

## Conclusion

The data indicate that the prevalence of overweight and obesity increased in Switzerland between 1992 and 2007, although the upward trends seemed to stabilize in the last five years. The persistent association between obesity and low educational level call for appropriately targeted interventions.

## Competing interests

The authors declare that they have no competing interests.

## Authors' contributions

PMV analysed the data and wrote part of the manuscript. PB and AC revised the manuscript critically for important intellectual content. FP revised the manuscript for important intellectual content and gave final approval of the version to be published. All authors read and approved the final manuscript.

## Pre-publication history

The pre-publication history for this paper can be accessed here:

http://www.biomedcentral.com/1471-2458/10/87/prepub

## Supplementary Material

Additional file 1 prevalence of reported obesity in Switzerland and other countries, by age group [43,44]. Click here for file

Additional file 2 prevalence of obesity in Switzerland and other countries, by age group Click here for file

Additional file 3 prevalence of obesity in Switzerland and other countries, by age group [45,46] Click here for file
