# Supplementary material for: Changes of overweight and obesity in the adult Swiss population according to educational level, from 1992 to 2007
Source: BMC Public Health. 2010 Feb 22;10:87. doi: 10.1186/1471-2458-10-87 (PMC2831837; doi:10.1186/1471-2458-10-87)
Supplement: Additional file 1 — prevalence of reported obesity in Switzerland and other countries, by age group [43,44]. [file 1471-2458-10-87-S1.DOC]

**Additional file 1a**: prevalence of reported obesity in Switzerland and other countries, by age group

|  | **Switzerland** | | | | **Portugal** | **France** | | | |
| --- | --- | --- | --- | --- | --- | --- | --- | --- | --- |
| Period | 1992-3 | 1997 | 2002 | 2007 | 2005-6 | 1997 | 2003 | 2006 | 2009 |
| Men |  |  |  |  |  |  |  |  |  |
| [18-34] | 3.3 | 4.2 | 4.5 | 4.9 | 6.7 | 5.0 ^a^ | 7.5 ^a^ | 7.5 ^a^ | 8.5 ^a^ |
| [35-44] | 5.4 | 4.7 | 6.5 | 7.8 | 13.0 | 7.5 | 11.0 | 11.5 | 13.0 |
| [45-54] | 7.8 | 8.0 | 11.6 | 10.3 | 22.0 | 11.5 | 14.5 | 15.0 | 16.0 |
| [55-64] | 11.3 | 11.5 | 12.7 | 13.4 | 22.1 | 15.0 | 18.0 | 19.0 | 20.0 |
| [65-74] | 9.3 | 11.0 | 13.1 | 13.3 | 19.7 | 12.0 | 15.0 | 17.0 | 18.0 |
| [75+ | 6.1 | 6.8 | 9.1 | 9.2 | 14.1 |
| Women |  |  |  |  |  |  |  |  |  |
| [18-34] | 1.7 | 3.0 | 4.0 | 4.6 | 7.3 | 6.0 ^a^ | 9.0 ^a^ | 10.0 ^a^ | 12.0 ^a^ |
| [35-44] | 3.9 | 4.9 | 5.9 | 6.0 | 13.0 | 8.0 | 12.0 | 15.0 | 14.5 |
| [45-54] | 5.5 | 6.8 | 8.5 | 8.7 | 20.5 | 10.0 | 14.0 | 15.0 | 16.0 |
| [55-64] | 8.6 | 12.7 | 9.8 | 10.8 | 24.6 | 12.0 | 14.0 | 18.0 | 19.5 |
| [65-74] | 8.7 | 13.0 | 12.2 | 13.1 | 23.9 | 10.5 | 15.5 | 16.0 | 18.0 |
| [75+ | 7.6 | 10.0 | 10.8 | 9.9 | 18.3 |

Obesity is defined as a BMI ≥30 kg/m^2^, obtained from self-reported height and weight. Data for Portugal obtained from [43] (responders only); data from France obtained from [44]. ^a^: for age group 25-34 years. Results are expressed in percentage.
