# Supplementary material for: Changes of overweight and obesity in the adult Swiss population according to educational level, from 1992 to 2007
Source: BMC Public Health. 2010 Feb 22;10:87. doi: 10.1186/1471-2458-10-87 (PMC2831837; doi:10.1186/1471-2458-10-87)
Supplement: Additional file 2 — prevalence of obesity in Switzerland and other countries, by age group [file 1471-2458-10-87-S2.DOC]

**Additional file 2**: prevalence of obesity in Switzerland and other countries, by age group

|  | **Switzerland** | | | | **England** | | |
| --- | --- | --- | --- | --- | --- | --- | --- |
| Period | 1992-3 | 1997 | 2002 | 2007 | 1993 | 1997 | 2002 |
| Men |  |  |  |  |  |  |  |
| [18-34] | 4.4 | 5.6 | 6.0 | 6.4 | 8.6 | 10.1 | 14.8 |
| [35-44] | 7.9 | 7.0 | 9.3 | 10.4 | 15.4 | 19.1 | 25.0 |
| [45-54] | 11.5 | 12.9 | 14.5 | 13.7 |
| [55-64] | 16.4 | 15.4 | 16.8 | 17.1 | 17.8 | 23.2 | 27.2 |
| [65-74] | 12.5 | 16.0 | 17.5 | 17.8 |
| [75+ | 7.7 | 12.1 | 13.0 | 11.7 | 10.8 | 11.9 | 19.2 |
| Women |  |  |  |  |  |  |  |
| [18-34] | 2.6 | 4.3 | 5.3 | 5.6 | 10.6 | 13.2 | 17.7 |
| [35-44] | 5.0 | 6.9 | 7.8 | 8.2 | 17.9 | 20.1 | 23.8 |
| [45-54] | 7.7 | 9.9 | 11.4 | 11.2 |
| [55-64] | 11.2 | 18.1 | 14.1 | 15.6 | 22.8 | 27.6 | 28.7 |
| [65-74] | 11.4 | 18.1 | 18.2 | 18.2 |
| [75+ | 10.4 | 13.5 | 14.7 | 14.1 | 15.8 | 21.9 | 22.3 |

Obesity is defined as a BMI ≥30 kg/m^2^, for corrected (Switzerland) or measured (England) data. Results are expressed in percentage. Data for England obtained from [5]. Swiss data was corrected for reporting bias using data from a previous study [12], i.e., adding 0.8 kg/m^2^ and 1.1 kg/m^2^ to the BMI values of men and women, respectively.
