# Supplementary material for: Changes of overweight and obesity in the adult Swiss population according to educational level, from 1992 to 2007
Source: BMC Public Health. 2010 Feb 22;10:87. doi: 10.1186/1471-2458-10-87 (PMC2831837; doi:10.1186/1471-2458-10-87)
Supplement: Additional file 3 — prevalence of obesity in Switzerland and other countries, by age group [45,46] [file 1471-2458-10-87-S3.DOC]

**Additional file 3**: prevalence of obesity in Switzerland and other countries, by age group

|  | **Switzerland** | | | | **Greece** | **Portugal** |
| --- | --- | --- | --- | --- | --- | --- |
|  | 1992-3 | 1997 | 2002 | 2007 | 1996-1999 | 2003-2005 |
| Men |  |  |  |  |  |  |
| [18-29] | 3.4 | 3.6 | 5.2 | 4.9 | 2.7 | 8.5 ^a^ |
| [30-39] | 6.5 | 7.5 | 7.8 | 8.7 | 10.0 | 17.4 |
| [40-49] | 10.1 | 10.2 | 11.6 | 12.5 | 16.1 | 16.8 |
| [50-59] | 14.6 | 13.2 | 17.2 | 14.7 | 15.9 | 22.0 |
| [60-69] | 13.4 | 17.8 | 16.7 | 18.3 | 15.2 | 14.9 ^b^ |
| [70+ | 10.5 | 12.6 | 14.5 | 14.5 | 10.6 | - |
| Women |  |  |  |  |  |  |
| [18-29] | 2.1 | 2.8 | 5.2 | 4.2 | 2.5 | 5.5 ^a^ |
| [30-39] | 3.8 | 5.7 | 6.1 | 8.3 | 8.2 | 13.3 |
| [40-49] | 6.2 | 8.8 | 10.0 | 8.7 | 12.1 | 17.2 |
| [50-59] | 9.6 | 13.1 | 13.7 | 13.9 | 18.8 | 24.2 |
| [60-69] | 12.2 | 19.6 | 15.1 | 15.8 | 24.3 | 24.2 ^b^ |
| [70+ | 10.4 | 15.2 | 16.2 | 16.7 | 17.2 | - |

Obesity is defined as a BMI ≥30 kg/m^2^, for corrected (Switzerland) or measured (Greece and Portugal) data. Results are expressed in percentage. Data for Greece obtained from [45]; data for Portugal obtained from [46]. Swiss data was corrected for reporting bias using data from a previous study [12], i.e., adding 0.8 kg/m^2^ and 1.1 kg/m^2^ to the BMI values of men and women, respectively. ^a^: 20-29 years only; ^b^: 60-64 years only.
